# Preparation, single-crystal structure and room-temperature phosphorescence of a covalent organic polymer containing Te–O–P bonds†

**DOI:** 10.1039/d4sc07210c

**Published:** 2025-02-17

**Authors:** Miaomiao Xue, Guigui Ye, Lei Zhang, Qiang Dong, Chun-Sing Lee, Zhen Li, Qichun Zhang

**Affiliations:** a Department of Materials Science and Engineering, City University of Hong Kong Hong Kong SAR P. R. China qiczhang@cityu.edu.hk; b Hubei Key Lab on Organic and Polymeric Opto-Electronic Materials, Department of Chemistry, Wuhan University Wuhan 430072 P. R. China lizhen@whu.edu.cn; c Department of Chemistry, Center of Super-Diamond and Advanced Films (COSDAF), Hong Kong Institute of Clean Energy (HKICE), City University of Hong Kong Hong Kong SAR P. R. China; d Shenzhen Research Institute, City University of Hong Kong Shenzhen Guangdong Province 518057 P. R. China

## Abstract

Room-temperature phosphorescent (RTP) single crystals of covalent organic polymers (COPs) are rarely reported due to the huge challenge in preparing single crystals from solutions as well as the difficulty in realizing RTP in metal-free organic molecules. Compared to other main group elements, tellurium is rarely successfully introduced into functional single-crystal COPs. Herein, we prepared colorless single crystals of a COP (CityU-21) through a reaction between tellurinyldibenzene and [1,1′-biphenyl]-4,4′-diyl-bis(phosphonic acid). Single crystal X-ray diffraction (SCXRD) analysis indicates that CityU-21 is a one-dimensional organic polymer through the covalent connection (Te–O–P bonds) between Te(Ph)_2_ moieties and [HO_3_P–Ph–Ph–PO_3_H] units. Due to the existence of one unreacted OH group in each phosphonic acid unit, multiple hydrogen-bonding interactions can be formed between adjacent polymer chains, which stabilizes the structure and promotes CityU-21 to form a pseudo-two-dimensional framework. Benefiting from the integrated effect of aromatic P=O parts, the heavy atoms (tellurium), and multiple hydrogen bonds, single crystals of CityU-21 display RTP behavior with a lifetime of 179 ms @ 540 nm and 158 ms @ 565 nm, a photoluminescence quantum yield of 84.69% and an afterglow time of 1.2 s. Moreover, CityU-21 can maintain its crystallinity and RTP character with an afterglow time of up to 0.8 s after immersion in different solvents for 60 hours, which can address the issue that most single crystals of RTP small molecules lose their crystallinity and RTP properties after solvent treatment.

## Abstract 2

CityU-21, a covalent organic polymer (COP), forms room-temperature phosphorescent (RTP) single-crystals with a stable pseudo-2D framework. It's the first RTP single-crystal COP and remains its crystallinity and RTP character in various solvents.

## Graphical abstract

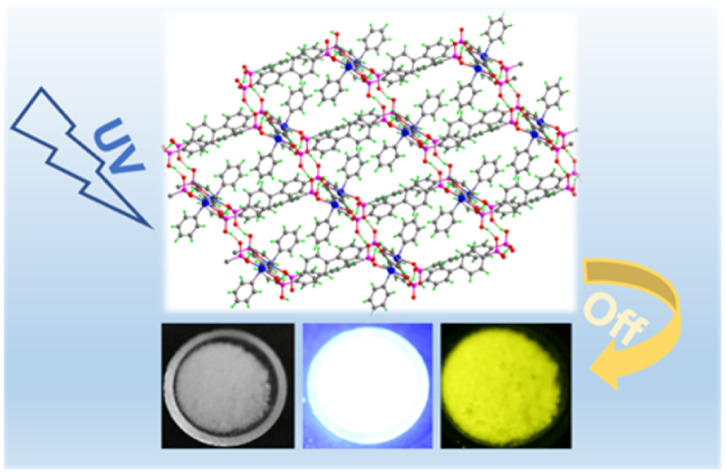

## Introduction

Recent advances in single-crystal structures of covalent organic polymers (COPs), including conjugated polymers and covalent organic frameworks (COFs),^1–6^ have a significant effect on the deep understanding of their structure–property relationship and the realization of high-performance devices,^7–11^ because these COPs have various applications in catalysis, separation, optoelectronics, energy storage, conversion, *etc.*^12–18^ However, COPs with the element tellurium, Te, in its backbone are rare due to the difficulty of Te to form covalent bonds in the backbone. On the other hand, there has been no success in room-temperature phosphorescent (RTP) single crystals of COPs, probably due to the following reasons: (i) direct preparation of single crystals of COPs from solutions is difficult and challenging because of the poor reversibility and high bonding energy of covalent bonds as well as the entanglements of COP chains; and (ii) phosphorescence lifetime and singlet–triplet intersystem crossing (ISC) depend on effective spin–orbit coupling (SOC); however, thermal motion and oxygen diffusion usually quench triplet excitons before phosphorescence generation occurring at room temperature (RT) in air, increasing the difficulty in the realization of RTP metal-free organic molecules. On the other hand, developing single crystals of COPs with RTP character is very important. Firstly, single crystals of COPs can allow us to determine their precise structures, stacking modes, interpenetration, and guest arrangements *via* single crystal X-ray diffraction (SCXRD) analysis, which provides us with more information related to the structure–property relationship and helps us to design/prepare various functional polymers for satisfying different demands and applications. Secondly, single crystals can restrict thermal motion and oxygen diffusion, which is favourable for RTP generation. In fact, RTP materials have been demonstrated to exhibit irreplaceable functionalization in optoelectronics, data security, biology, *etc.*^19–25^ Although considerable progress has been made in RTP single crystals of small molecules,^26–30^ the research on RTP single crystals of COPs is rare. Moreover, due to their rigid and stable backbone, COPs could be an alternative strategy to address solvent instability of RTP single crystals based on most small-molecule crystals.

Here, we designed and prepared RTP single crystals of a COP containing Te–O–P bonds (named CityU-21) by introducing heavy atoms (tellurium) and [P=O] groups into the backbone of CityU-21 to prompt the SOC process. SCXRD analysis indicates that in the structure of CityU-21, biphenyl units are covalently bonded with Te(Ph)_2_ moieties *via* [HPO_3_] parts to form a one-dimensional (1D) covalent organic polymer, and multiple hydrogen-bonding interactions between each adjoining polymer chain prompt the formation of a pseudo-two-dimensional (2D) framework. Because the heavy-atom (Te) effect in CityU-21 can generate a more significant amount of SOC to facilitate the ISC process, and the strong hydrogen bonding interaction can enhance the stability and rigidity of its structure to suppress the energy loss of the non-radiative transition, single crystals of CityU-21 show excellent RTP performance with a lifetime of 179 ms @ 540 nm and 158 ms @ 565 nm. The photoluminescence quantum yield (PLQY) can reach 84.69%, and the afterglow time is 1.2 s. Moreover, even after the treatment with common solvents (water, hexane (hex), toluene (Tol), dichloromethane (DCM), and dimethylformamide (DMF)), single crystals of CityU-21 displayed high solvent stability and maintained their crystallinity as well as their RTP character with an afterglow time of up to 0.8 s. This research can provide a new synthetic strategy for designing and preparing single crystals of novel COPs, enrich the family of COPs and broaden their diverse applications.

## Results and discussion

**Fig. 1:**
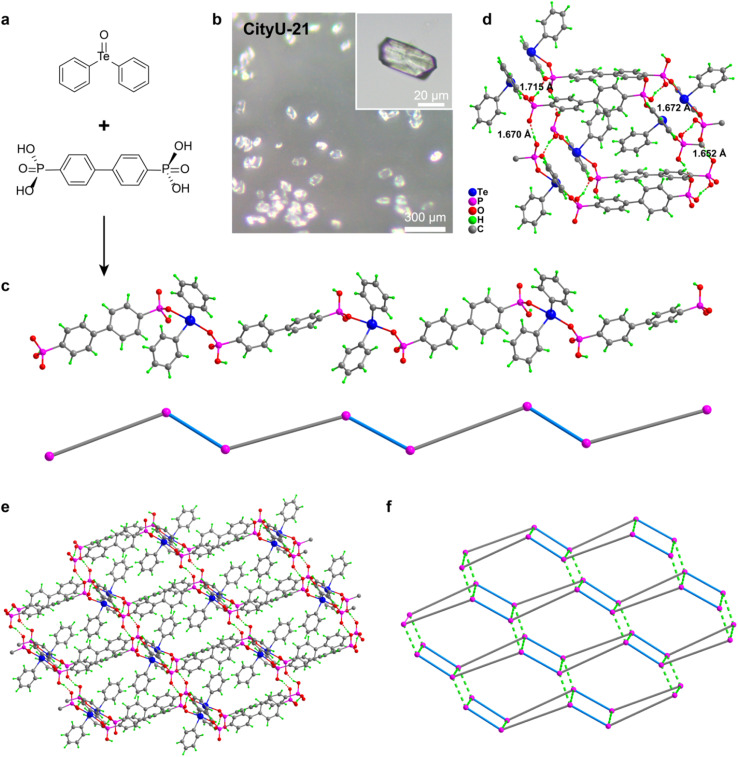
(a) Preparation of single crystals of CityU-21. (b) Optical microscopy image for single-crystal CityU-21. (c) The 1D polymer chain of CityU-21 and simplified 1D structure diagram (phosphonic acid ligands in gray and Te(Ph)_2_ in blue). (d) The multiple hydrogen bonding interactions between adjacent chains in CityU-21. (e) Pseudo-2D single-crystal structure of CityU-21. (f) Simplified hydrogen-bonded pseudo-2D structure diagram (phosphonic acid ligands in gray, Te(Ph)_2_ in blue and hydrogen bond interactions in green).

The colourless single crystals of CityU-21 were formed after a reaction between tellurinyldibenzene and [1,1′-biphenyl]-4,4′-diyl-bis(phosphonic acid) in a mixture of ethanol, 1,4 dioxane, and acetic acid at 100 °C for two days *via* a solvothermal method (Fig. 1a). The size of single crystals is in the range of 60–90 μm (Fig. 1b). SCXRD analysis indicates that CityU-21 belongs to the space group of *P̄*1 (*a* = 9.41547(15) Å, *b* = 10.53235(17) Å, and *c* = 23.2502(2) Å, Tables S1–S3†) with the asymmetric unit containing two Te(phenyl)_2_ units and two [1,1′-biphenyl]-4,4′-[(PO_2_)OH]_2_ (Fig. S1†). As described in Table S2,† bond lengths of HO–P (1.545–1.555 Å) and O=P (1.489 and 1.496 Å) help to distinguish and confirm these positions of hydrogen atoms in four [HPO_3_] units. One OH group from each PO_3_H_2_ group in the molecule of [1,1′-biphenyl]-4,4′-diylbis (phosphonic acid) reacted with tellurinyldibenzene to form a 1D COP (Fig. 1c), and the rest of the OH groups remained unreacted. Strong hydrogen bonds (OH⋯O=P) with a distance of 1.652–1.715 Å as shown in Fig. 1d and Table S4,† were formed between these unreacted OH groups from each polymer chain and the P=O units in adjoining polymer chains. These multiple hydrogen-bonding interactions prompt CityU-21 to become a hydrogen-bonded pseudo-2D framework (Fig. 1e, f and S2, S3†). Accordingly, these unreacted OH groups further stabilize the structure of CityU-21 and restrict the vibration and movement of polymer chains.

**Fig. 2:**
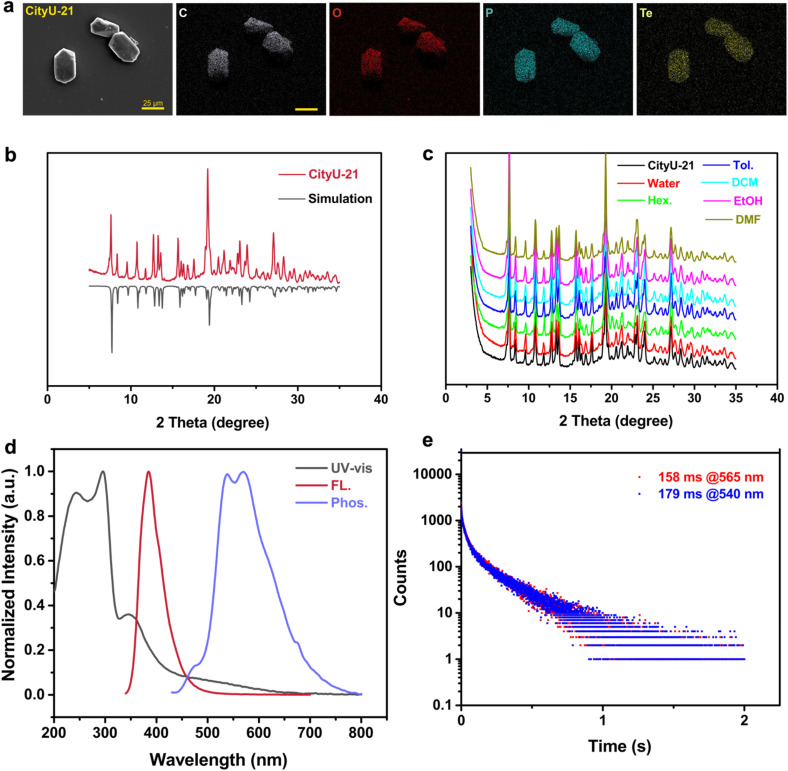
(a) The SEM image and the corresponding mapping images of C (gray), O (red), P (green), and Te (yellow) elements for single crystals of CityU-21. (b) PXRD patterns of CityU-21 and the simulated curve from single crystals. (c) The PXRD curves of single crystals of CityU-21 immersed in different solvents for 60 hours. (d) UV-vis absorption (UV-vis), fluorescence (FL), and phosphorescence (Phos.) spectra of single crystals of CityU-21 at room temperature in air (*λ*_ex, PL._ = 330 nm and *λ*_ex, Phos._ = 350 nm). (e) Phosphorescence decay curves of single crystals of CityU-21 at room temperature under ambient conditions (*λ*_ex, Phos._ = 350 nm).

Scanning electron microscopy (SEM) and elemental mapping, as well as Fourier-transform infrared spectroscopy (FTIR) analysis, were used to further confirm the structure of CityU-21. SEM images and the corresponding elemental mappings suggest that single crystals of CityU-21 have a quasi-cuboid shape containing the elements of carbon, oxygen, phosphorus, and tellurium (Fig. 2a). As exhibited in Fig. S4,† the peaks in the range of 790–580 cm^−1^ result from the stretching of Te–O bonds,^31^ while the peaks at 960–840 cm^−1^ are a result of P–O bonds in CityU-21.^32^ As presented in Fig. 2b, the experimental powder X-ray diffraction (PXRD) data matched well with the simulated pattern from SCXRD data of CityU-21, implying its high phase purity. In addition, to confirm the solvent stability of single crystals of CityU-21, these crystals were immersed for 60 hours in different solvents including water, hexane, toluene, dichloromethane, and dimethylformamide (Fig. S5†). As shown in Fig. 2c, all solvent-treated CityU-21 samples maintained their original crystallinity, indicating their high stability in different solvents. Furthermore, the thermal stability of CityU-21 was studied *via* thermogravimetric analysis (TGA, Fig. S6†). CityU-21 could be stable up to 327 °C considering a 5% weight loss, suggesting its high thermal stability. The excellent solvent/thermal stability is essential for the subsequent applications of single crystals of CityU-21.

**Fig. 3:**
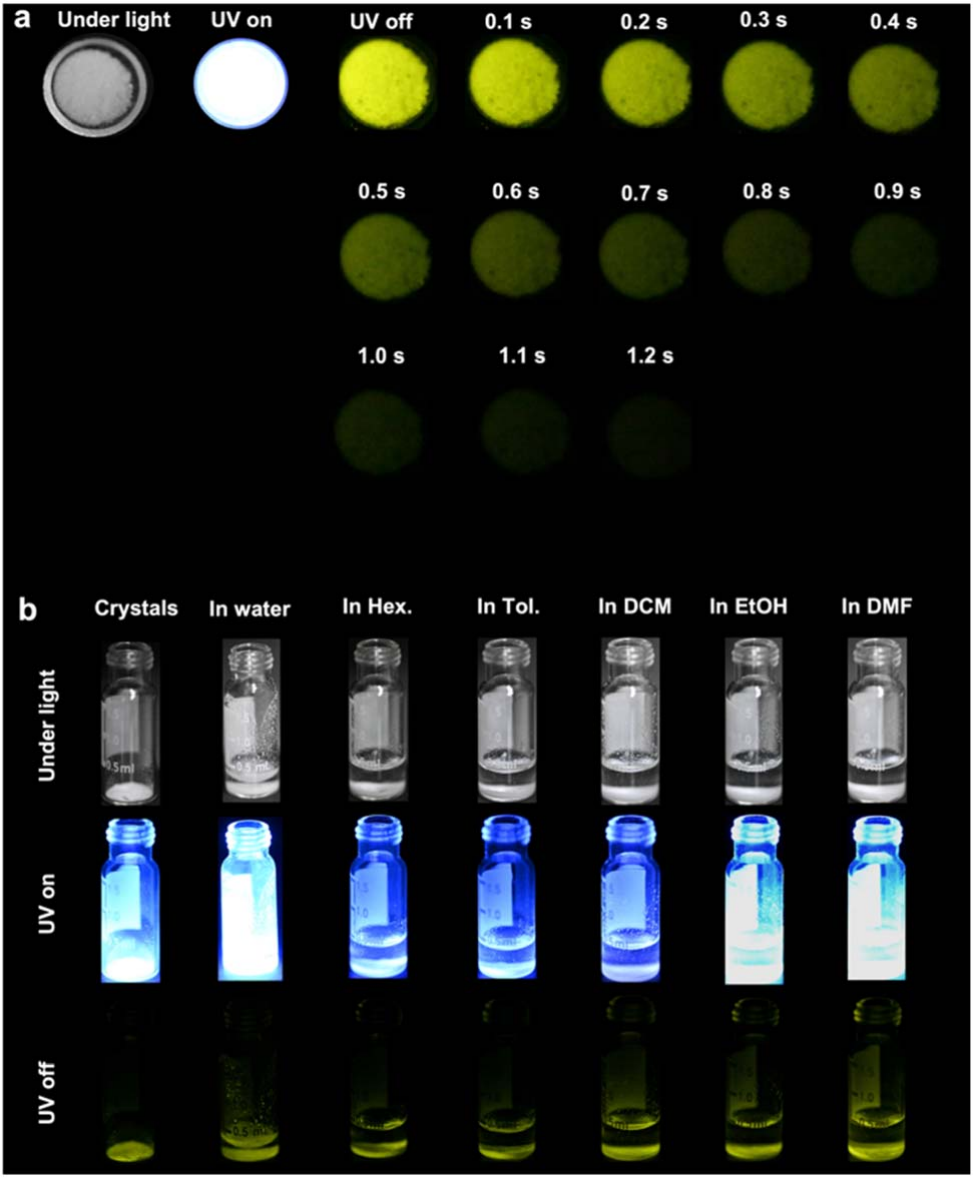
(a) The photographs of single crystals of CityU-21 before and after switching off 365 nm UV light. (b) The photographs of single crystals of CityU-21 treated with/out solvents before and after switching off 365 nm UV light.

The optical properties of single crystals of CityU-21 were studied using the UV-vis absorption spectrum, the fluorescence spectrum, and the phosphorescence spectrum at RT in air (Fig. 2d). There are three pronounced UV-vis absorption peaks, where two short-wavelength absorption peaks at 240–300 nm can be assigned to the π–π* transition from the biphenyl units and phenyl units in CityU-21, and the longer wavelength peak at 350 nm can be ascribed to the possible charge transfer (CT) interactions between [HO_3_P–Ph–Ph–PO_3_H] units and Te(Ph_2_) species. Apart from the fluorescence peak at 388 nm, single crystals of CityU-21 emit intense phosphorescence at room temperature under ambient conditions with two peaks at 540 nm and 565 nm, respectively, with a PLQY of 84.69%. Furthermore, CityU-21 had been demonstrated to show long lifetimes of 179 ms @ 540 nm and 158 ms @ 565 nm at RT in air from the phosphorescence decay curves (Fig. 2e). These results indicate that CityU-21 has RTP properties, which was further evidenced by photographs in Fig. 3a and Movie S1.† The afterglow time of single crystals of CityU-21 is 1.2 s.

**Fig. 4:**
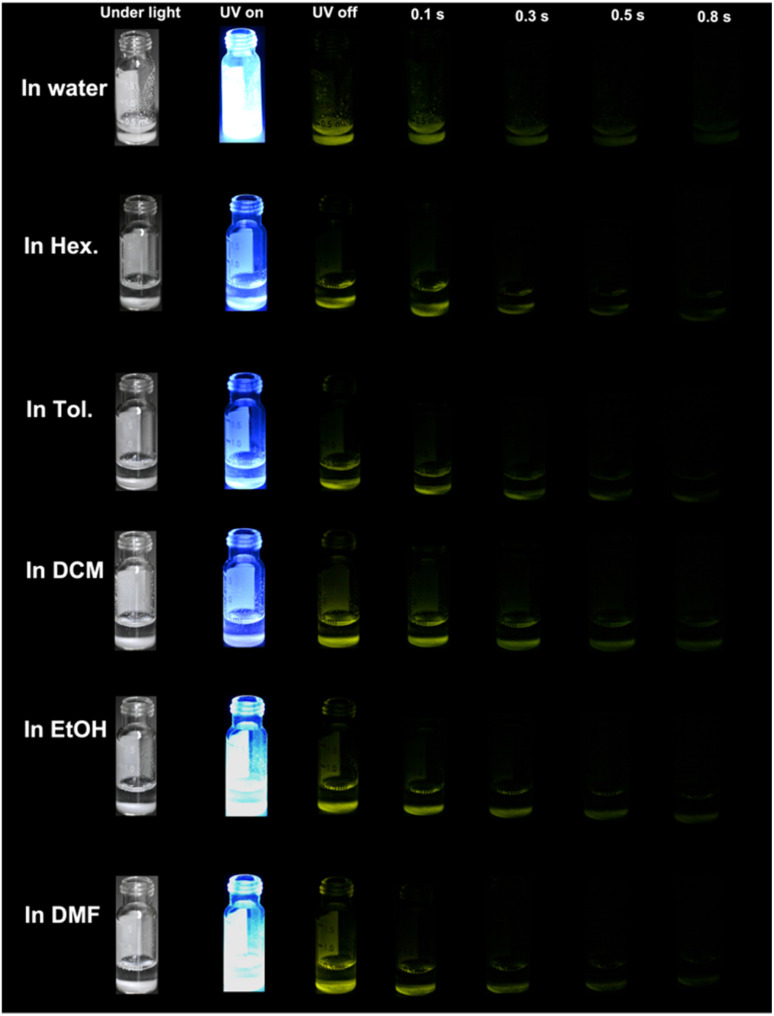
The photographs of single crystals of CityU-21 immersed in different solvents with RTP properties.

The possible reasons that CityU-21 has an RTP character can be analyzed as follows. Firstly, the P=O parts in the backbone can exhibit some degree of spin–orbit coupling, prompting intrinsic triplet generation through ISC. Secondly, the heavy atom (tellurium) in CityU-21 enhances the mixture of the singlet and triplet states of excited excitons and singlet–triplet conversion. Thirdly, multiple hydrogen bonding effects not only make adjacent polymer chains close enough to form a relatively rigid hydrogen-bonded pseudo-2D structure and stabilize the whole structure, but also suppress nonradiative decay and decrease energy loss. Furthermore, due to the integrating effect of P=O parts in the backbone, the heavy atom effect and hydrogen bonds, single crystals of CityU-21 display RTP in air and can maintain their RTP character in most solvents. Fig. 3b and 4 show that single crystals of CityU-21 can sustain RTP phenomena with an afterglow time up to 0.8 s when soaked in different polar solvents. Besides, their structure and crystallinity remained unchanged after being immersed in different solvents for 60 h, confirmed by the PXRD curves in Fig. 2c. The high stability and good RTP behaviour of CityU-21 in solvents can solve the dilemma that single crystals of RTP small molecules usually lose their crystalline state and RTP properties after solvent treatment, which can broaden the application of RTP materials.

## Conclusion

In summary, different from traditional COPs, we adopted a heavy element (tellurium) from main group VI to construct the polymer backbone and successfully obtained single crystals of a COP (CityU-21), where metal-free organic ligands are covalently connected together *via* Te–O–P bonds to form polymer chains. The unreacted OH groups in each polymer chain allow the formation of multiple hydrogen bonding interactions with neighboring chains to produce a hydrogen-bonded pseudo-2D framework. The combined effects from P=O units, the heavy atoms (Te), and multiple hydrogen bonds endow CityU-21 with strong RTP emission at 540 nm and 565 nm with a PLQY of 84.64%. The phosphorescence lifetime can be as long as 179 ms at 540 nm and 158 ms at 565 nm with an afterglow time of 1.2 s. This is the first example to report RTP character in single crystals of COPs. Furthermore, after immersion in different solvents for 60 hours, the RTP character and crystallinity of single crystals of CityU-21 can be still maintained with an afterglow time of up to 0.8 s, which can solve the dilemma that single crystals of most small molecules lose their crystallinity and RTP character after solvent treatment. This work expands the variety of COPs, provides an alternative synthetic strategy for the construction and preparation of single crystals of COPs, broadens applications of COPs, and provides another pathway to design and develop RTP materials.

## Data availability

All experimental procedures and methods are listed in the ESI.† The crystallographic data for this work have been deposited at the Cambridge Crystallographic Data Centre (CCDC) with CCDC 2339864. These data can be obtained free from the Cambridge Crystallographic Data Centre *via*www.ccdc.cam.ac.uk/data_request/cif.

## Author contributions

M. Xue, G. Ye, and L. Zhang contributed equally.

## Conflicts of interest

The authors declare no competing financial interest.

## Supplementary Material

SC-OLF-D4SC07210C-s001

SC-OLF-D4SC07210C-s002
