# Supplementary material for: Preparation, single-crystal structure and room-temperature phosphorescence of a covalent organic polymer containing Te–O–P bonds
Source: Chem Sci. 2025 Feb 17;16(12):5260–5. doi: 10.1039/d4sc07210c (PMC11848404; doi:10.1039/d4sc07210c)
Supplement: SC-016-D4SC07210C-s001 [file SC-016-D4SC07210C-s001.pdf]

Supplementary Information (SI) for Chemical Science.
This journal is © The Royal Society of Chemistry 2025

Supporting Information

Preparation, single-crystal structure and room-temperature phosphorescence of a covalent organic polymer containing Te-O-P bonds

Miaomiao Xue,[a,†] Guigui Ye,[b,†] Lei Zhang,[a,†] Qiang Dong,[a] Chun-Sing Lee,[c] Zhen Li,[b,*] Qichun Zhang[a,c*]

Materials and Methods

All starting chemicals were commercially available and used without further purification. All solvents were purchased from Anaqua (Hong Kong) Company Limited.

NMR data was collected on the Bruker Avance-400 spectrometers (400 MHz for ^{1}H, 101 MHz for ^{13}C). Chemical shifts (δ) were reported in parts per million (ppm) with the following abbreviations to describe peak splitting patterns (*m* = multiple). TMS was used as an internal standard.

Single crystal X-ray diffraction (SCXRD) characterization was performed on the Rigaku X-ray Single Crystal Diffractometer System (Rigaku SmartLab 9kW-Advance) at 150 K. The topological analysis was carried out on the TOPOS program. PXRD was conducted on Rigaku X-ray Diffractometer SmatlabTM 9kW at room temperature.

The Fourier transform infrared (FTIR) spectrum from 4,000 to 400 cm^{-1} was collected on a Perkin Elmer Spectrum II. Scanning electron microscope (SEM) and elemental mappings were carried out on the Thermo Fisher Quattro S Environmental SEM. Thermal gravimetric analyses (TGA) data was measured on a Perkin-Elmer Simultaneous Thermal Analyzer STA 6000 under nitrogen flow (20 mL/min) with a heating rate of 10 °C min^{-1}.

UV-vis spectra in the range of 200-800 nm were performed on the Hitachi UH4150 UV-VIS-NIR Spectrophotometer. Optical photos of these crystals were taken by the Zeiss microscope. Photoluminescence, phosphorescence spectra, lifetimes, and quantum yield of single crystals of CityU-21 were determined on an FLS980 spectrometer at room temperature in the air. During the quantum yield test, the excitation range was 335 to 360 nm, while the luminescence range was collected from 360 nm to 700 nm. The photos of RTP phenomena for single crystals of **CityU-21** were taken by Nikon Z9.

The video for recording the RTP phenomena of single crystals of **CityU-21** is supplied as Movie S1 in a separate file.

Synthesis

Synthesis of monomer

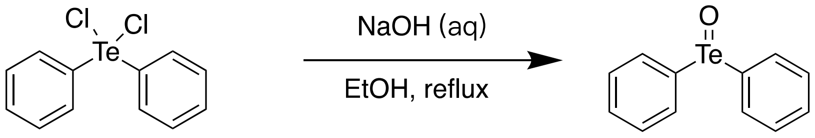

Tellurinyldibenzene was synthesized according to our previously published paper.[1] ^{1}H NMR (400 MHz, d_4-MeOH): δ (ppm) 7.55-7.62 (m, 6H), 7.88-7.93 (m, 4H); ^{13}C NMR (400 MHz, d_4-MeOH): δ (ppm) 134.7, 132.3, 130.9, 129.3.

Synthesis of CityU-21

Tellurinyldibenzene (15.0 mg, 0.05 mmol), [1,1'-biphenyl]-4,4'-diylbis (phosphonic acid) (6.5 mg, 0.021 mmol) and 160 μL acetic acid dissolved in a mixture of 0.7 ml of absolute ethanol and 0.2 mL of 1,4-dioxane were added to a glass ampoule. After ultrasonication, the glass ampoule was frozen in an aqueous liquid nitrogen bath, evacuated three times, and then sealed under a vacuum atmosphere. After heating at 100 °C for 2 days, large-size colorless crystals were formed. Then, the glass ampoule was cooled to room temperature, and these crystals were collected by filtration. These crystals were further washed with methanol, 1,4-dioxane, deionized water, and ethanol in turn. Then, these crystals were dried under a vacuum to afford colorless **CityU-21** (14.9 mg, 72% yield).

Table S1. Crystal data and structure refinement of **CityU-21**.

	**CityU-21**
**Empirical formula**	$C_{48}H_{40}O_{12}P_4Te_2$
**Formula weight**	1187.88
**Crystal system**	triclinic
**Space group**	*P*-1
a **(Å)**	9.41547(15)
b **(Å)**	10.53235(17)
c **(Å)**	23.2502(2)
α **(°)**	93.7232(11)
β **(°)**	99.2727(11)
γ **(°)**	91.1692(13)
V **(Å^3)**	2269.61(6)
Z	2
D_{calc} **(g·cm^{-3})**	1.738
**Abs.coeff.(mm^{-1})**	12.029
***F*(000)**	1176.0
**Reflns collected**	24255
**GOFon** F^2	1.056
R_1[a]	0.0244
wR_2**(all data)**[b]	0.0586
**CCDC number**	2339864

Table S2. Selected bond distance (Å) for **CityU-21**.

**Atom1**	**Atom2**	**Length/Å**
Te1	O1[1]	2.1181(18)
Te1	O8	2.1126(18)
Te1	C10	2.130(3)
Te1	C16	2.123(3)
Te2	O2	2.0891(18)
Te2	O4	2.1019(18)
Te2	C2	2.125(3)
Te2	C7	2.134(3)
P1	O2	1.5552(19)
P1	O7	1.489(2)
P1	O11	1.556(2)
P2	O8	1.5470(19)
P2	O9	1.503(2)
P2	O12	1.537(2)
P3	O1	1.5457(19)
P3	O5	1.549(2)
P3	O10	1.496(2)
P4	O3	1.499(2)
P4	O4	1.5461(19)
P4	O6	1.5515(19)

[1]-1+X,-2+Y,-1+Z

Table S3. Selected bond angles (°) for **CityU-21**.

Atom1	Atom2	Atom3	Angles (°)
O1[1]	Te1	C10	85.59(9)
O1[1]	Te1	C16	84.56(9)
O8	Te1	O1[1]	167.95(7)
O8	Te1	C10	86.30(9)
O8	Te1	C16	88.34(9)
C16	Te1	C10	101.44(11)
O2	Te2	O4	166.41(8)
O2	Te2	C2	87.89(9)
O2	Te2	C7	84.91(9)
O4	Te2	C2	84.89(9)
O4	Te2	C7	85.87(9)
C2	Te2	C7	105.04(11)
C17	C7	Te2	118.2(2)
C35	C7	Te2	121.0(2)
C30	C10	Te1	119.2(2)
C32	C10	Te1	119.8(2)

[1]-1+X,-2+Y,-1+Z

Table S4. Selected Hydrogen Bond Lengths (Å) and Bond Angles (°) in CityU-21.

D H A	d (D-H)/ Å	d (H-A)/ Å	d (D-A)/ Å	D-H-A/ °
O5 H5 O9[1]	0.84	1.670	2.469(3)	157.7
O6 H6 O7[2]	0.84	1.672	2.495(3)	166.6
O11 H1 O3[1]	0.84	1.715	2.498(3)	154.3
O12 H12 O10[3]	0.84	1.652	2.441(3)	155.5

[1]1-X,1-Y,1-Z; [2]+X,-1+Y,+Z; [3]-1+X,-1+Y,-1+Z; [4]-1+X,-2+Y,-1+Z

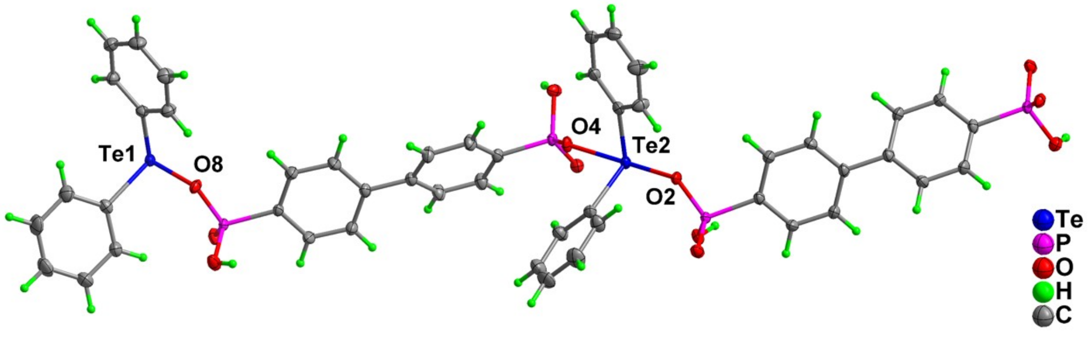

Figure S1. The asymmetric unit of **CityU-21**. Thermal ellipsoids are drawn at the 50% probability level.

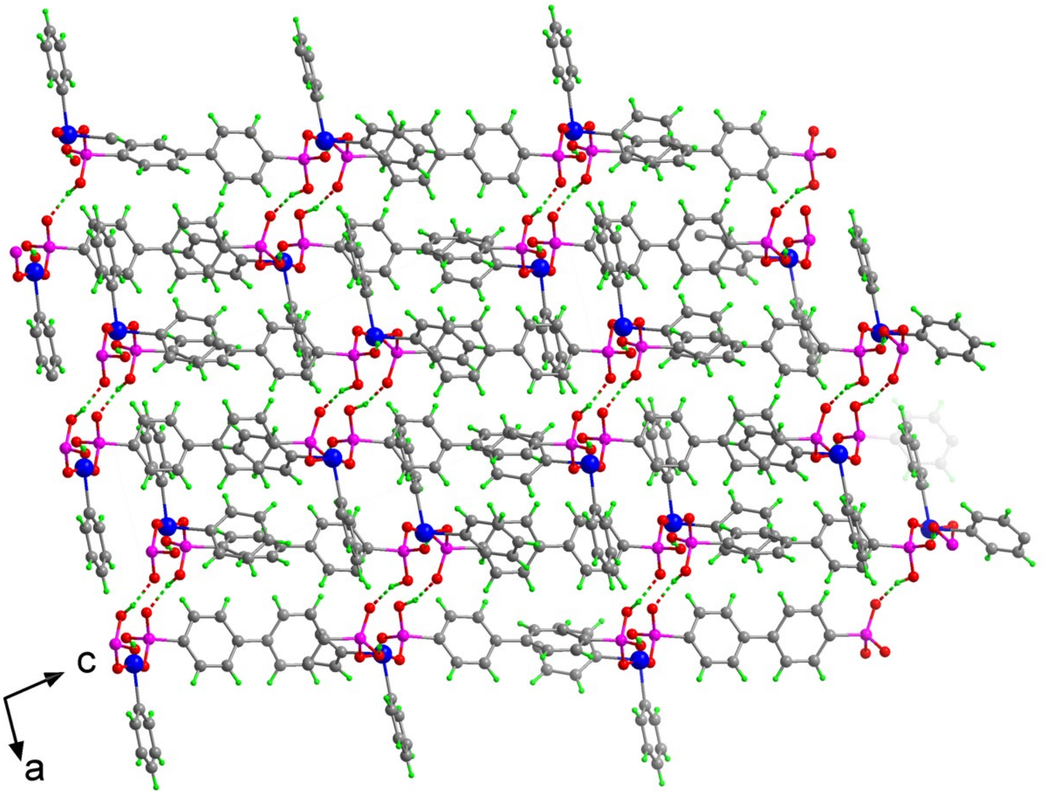

Figure S2. View of the hydrogen-bonded 2D supramolecular layer structure of **CityU-21** based on the chains along the *b* axis. Te, blue; C, gray; O, red; P, pink; H, green.

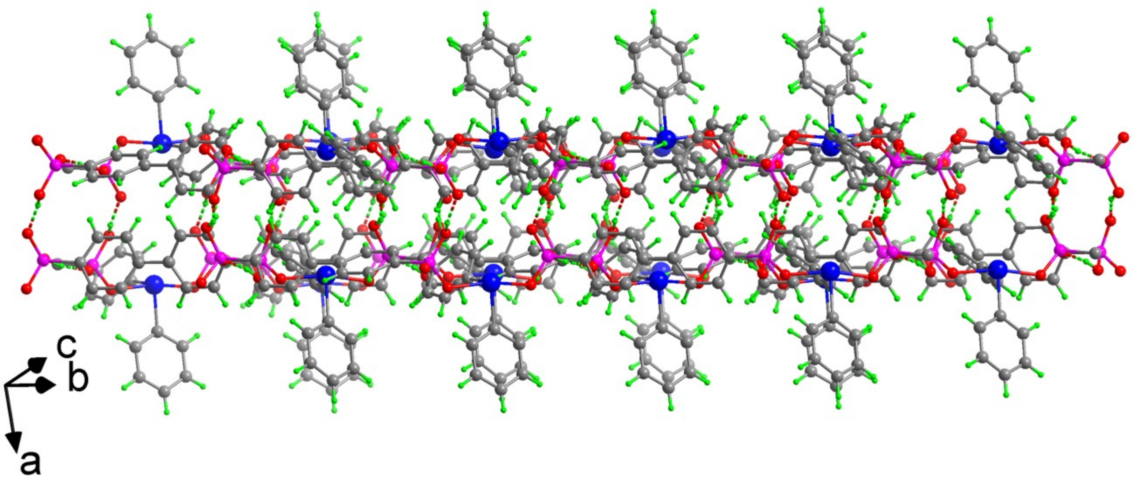

Figure S3. View of the hydrogen-bonded 2D supramolecular layer structure of **CityU-21**. Te, blue; C, gray; O, red; P, pink; H, green.

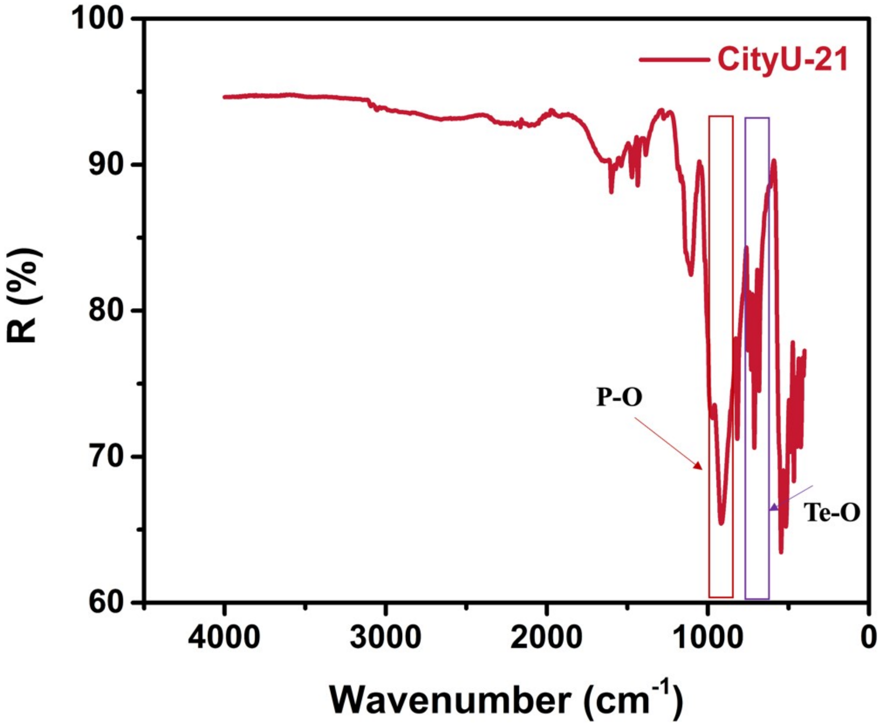

Figure S4. FTIR spectrum of **CityU-21**.

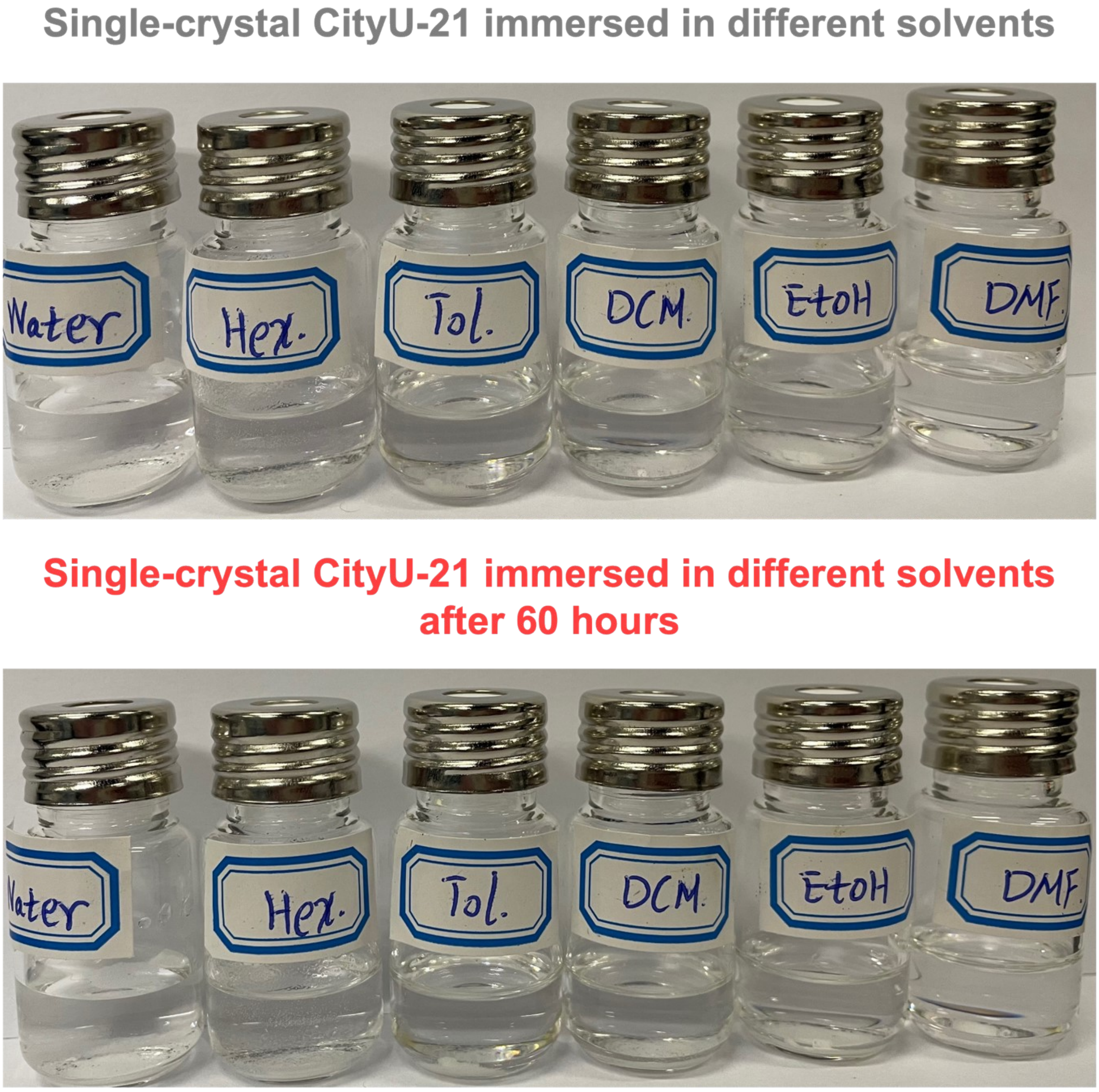

Figure S5. Single crystals of **CityU-21** were soaked in different solvents (hexane (Hex.), toluene (Tol.), dichloromethane (DCM), ethyl alcohol (EtOH), dimethylformamide (DMF)).

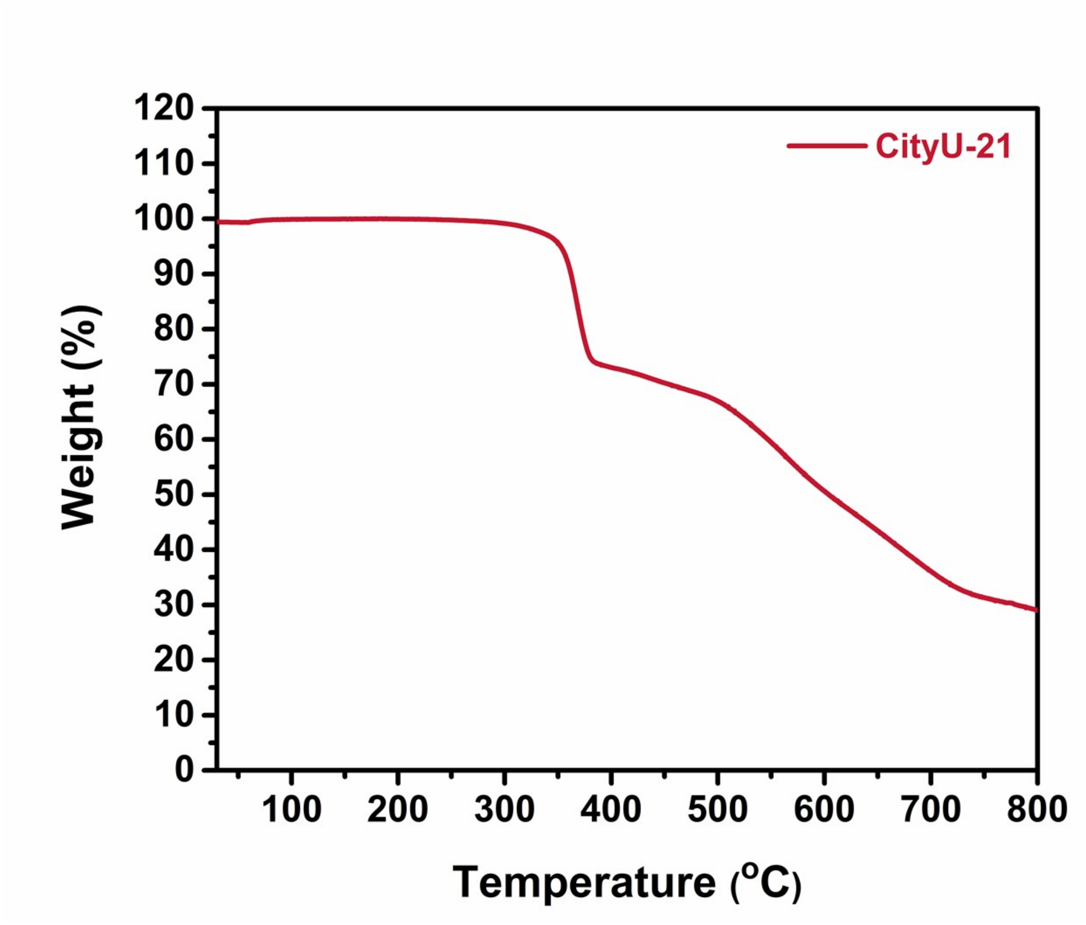

Figure S6. TGA curve of single crystals of **CityU-21**.

Reference

1. M. Xue, L. Zhang, X. Wang, Q. Dong, Z. Zhu, X. Wang, Q. Gu, F. Kang, X.-X. Li and Q. Zhang, *Angew. Chem. Int. Ed.*, 2024, **63**, e202315338.
